# Editorial Note: A Meta-Analysis of Studies of Treatments for Feline Urine Spraying

**DOI:** 10.1371/journal.pone.0342540

**Published:** 2026-02-10

**Authors:** The PLOS One Editors

The *PLOS One* Editors have identified concerns about this article’s [1] peer review. In light of this issue, readers are advised to interpret the article [1] with caution. We regret that this was not addressed prior to the article’s publication.

The Editors have no evidence of any author involvement in the peer review concerns.
